# Balancing act: An autoethnographic study of one medical educator's first year as a mentor

**DOI:** 10.1002/ase.70110

**Published:** 2025-08-13

**Authors:** Andrew S. Cale

**Affiliations:** ^1^ Department of Anatomy, Cell Biology, & Physiology Indiana University School of Medicine Indianapolis Indiana USA

**Keywords:** autoethnography, medical education, mentoring, professional identity formation, transformative learning theory

## Abstract

Novice faculty mentors often struggle with the transition from mentee to mentor. Although they may face similar challenges, each mentor's experience and journey of professional identity formation is unique, influenced by their background, experiences, relationships, and context. This autoethnographic study describes my personal experiences as a first‐year faculty mentor in medical education, including the challenges I encountered, lessons I learned, and recommendations I have for novice faculty mentors in similar situations. Between January and August 2024, I recorded a series of reflective audio diaries after mentor‐mentee project meetings. Each audio diary followed a semi‐structured format and included my description of events, critical reflections, and mentoring plans. I then performed reflexive thematic analysis on the audio diaries using both deductive codes based on Transformative Learning Theory and inductive codes derived from the collected data. Through this analysis, I generated the overarching theme of *The Balancing Act*, in which faculty mentors must constantly balance an ever‐shifting collection of competing factors in their mentoring relationships (e.g., time, effort, contributions, and expectations). This theme also included five sub‐themes: (i) *Balancing Project Contributions*, (ii) *Balancing Time and Obligations*, (iii) *Balancing Oversight and Autonomy*, (iv) *Shifting the Balance*, and (v) *Communicating the Balance*. Although I found *The Balancing Act* challenging to achieve in my first year, clear and consistent communication greatly reduced the difficulty. With continued practice and reflection, I believe my skills in navigating *The Balancing Act* will improve further, allowing me to support mentees more effectively.

## INTRODUCTION

Faculty members often mentor junior colleagues such as graduate students and postdoctoral scholars as part of their academic responsibilities. Although faculty members spend years developing the knowledge, skills, and experience required to perform independent research themselves, learning how to guide a trainee through the same process can present a unique challenge. Novice mentors often struggle with clear communication, setting expectations, conflict resolution, and maintaining professional boundaries as they transition from the role of mentee to that of the mentor.^1^ Although faculty sometimes face similar challenges with mentorship, each faculty mentor's experience is “complex and multifaceted, with each relationship requiring a constellation of different attributes”.^2^ In this autoethnographic study, I will detail my own unique experience as a first‐year faculty mentor in medical education to highlight the challenges that inexperienced mentors face as they transition into their new role. In doing so, I hope that these narratives will offer guidance for supporting novice mentors and express my solidarity with fellow mentors who may be facing similar challenges.

### Mentoring and mentor development

Mentoring is described as a complex two‐way relationship between a mentor and mentee, the primary goal of which is the education and growth of the mentee, although oftentimes both parties experience personal and professional growth.^3^, ^4^, ^5^ Although mentoring involves the transfer of knowledge and skills to a trainee, the expertise and teaching ability of the educator do not necessarily equate to mentoring ability.^3^ In many cases, successful faculty members may serve as poor mentors due to their disposition, time and resource constraints, or lack of motivation.^3^, ^6^, ^7^ A faculty mentor's socio‐cultural characteristics and perspectives may also negatively impact the quality of their mentorship.^8^, ^9^ Some faculty members may find it challenging to mentor students with identities that differ from their own (e.g., race, ethnicity, gender, sexual orientation, or religious affiliation), especially if they hold biases toward that identity group.^8^, ^9^ Conversely, effective mentors consistently demonstrate several key characteristics and behaviors: actively listening to mentees, acting as a guide rather than an enforcer, making themselves available and accessible, being supportive and caring, providing practical advice, giving honest and constructive feedback, developing mutual respect, exhibiting curiosity but also discretion, being capable of self‐reflection, and communicating openly and honestly.^5^

Mentoring, much like teaching, is not an inherent trait but rather a skill that requires intentional training and practice.^3^, ^10^, ^11^ However, faculty members often receive little to no training on how to provide effective mentorship.^3^, ^11^ Instead, many new mentors default to mentoring trainees in a manner similar to their own mentor's—for better or worse.^3^ If the new faculty mentor received positive, supportive mentorship, they may adopt a similar mentoring style, leading to a productive, mutually beneficial relationship with their mentees.^3^ However, in the worst‐case scenario, the novice faculty mentor, whether intentionally or not, may perpetuate an absent, abusive, or unequal mentoring style and neglect the expectations, needs, and goals of their mentee.^3^ In either situation, the new mentor emulates the mentoring style of another, rather than developing one that leverages their own strengths or incorporates evidence‐based techniques. For inexperienced faculty mentors interested in developing their mentoring abilities, peer‐reviewed literature and professional development resources (e.g., trainings, workshops, and webinars) are often available through their home institutions or professional organizations.^3^ However, meaningful engagement in these resources often requires a significant investment of time and effort and detracts from the new mentor's other responsibilities.^3^ Regardless of how a new faculty mentor decides to improve their mentoring abilities, either through trial‐and‐error, external support, or both, intentional engagement and critical self‐reflection play a major role.^3^, ^12^ Such reflective processes transform the new mentor's behaviors, mindsets, and perspectives, allowing them to shape and adjust their mentoring style as needed.

As faculty mentors engage in mentoring and develop their abilities, they transition from *doing* the work of a mentor to *being* a mentor, indicating an evolution of their professional identity. The process of professional identity formation (PIF) involves the development of the professional values, morals, aspirations, and behaviors essential to a particular profession.^13^, ^14^ Key factors such as experiential learning, critical reflection, formative feedback, guidance from role models and mentors, and a community of fellow learners commonly drive the PIF process.^14^, ^15^, ^16^ However, each individual experiences the PIF process differently, with some seamlessly incorporating their new identity and others grappling with the dissonance between their current and new identities.^13^, ^17^ The PIF process can also involve periods of growth and progress, along with occasional stagnation or regression.^13^, ^17^ Although much of the PIF process emphasizes the individual, it does not occur in a vacuum and requires socialization into the professional community.^13^, ^14^, ^18^ By interacting with role models, mentors, and the community, the individual learns the norms of the profession.^13^ Through this process, faculty members learn to embody mentors and integrate into their community of practice.

### Theoretical framework

In this study, I employ Transformative Learning Theory (TLT) as the lens through which I will explore my development and professional identity formation as a mentor. This theory details several key stages in the process of adult learning and aligns well with professional identity formation. Much like PIF, TLT emphasizes that adult learners build on their existing knowledge by making sense of new experiences through critical self‐reflection and dialogue with others.^14^, ^15^, ^19^ In TLT, a disorienting dilemma catalyzes the learning process and pushes the learner to reevaluate their identity, behaviors, and understanding of the world.^19^ Learning follows a 10‐step process: (1) encountering or grappling with a disorienting dilemma, (2) self‐examination with feelings of fear, anger, guilt, or shame, (3) a critical assessment of assumptions, (4) recognition that one's discontent and the process of transformation are shared, (5) exploration of options for new roles, relationships, and action, (6) planning a course of action, (7) acquiring knowledge and skills for implementing one's plans, (8) provisional trying of new roles, (9) building competence and self‐confidence in new roles and relationships, and (10) a reintegration into one's life on the basis of conditions dictated by one's new perspective.^19^ Progression through these steps can vary greatly. Learning may occur over an extended period through steady, incremental changes to the individual's perspective and behaviors.^19^ Conversely, a single profound, revelatory experience may cause a sudden and dramatic shift in the individual's worldview.^19^ Regardless of the specific path, by the end of the process, the individual has made meaning of their disorienting experiences through self‐reflection and discourse, leading to new perspectives and behaviors.^19^

### Autoethnography

Given the uniqueness of every individual's mentor identity and journey through professional identity formation, autoethnography serves as the ideal methodology for me to study my own development as a mentor. Autoethnography is a reflexive form of qualitative research in which a researcher combines autobiography and ethnography to explore and analyze personal experiences related to a particular culture or group.^20^ It was pioneered in the 1970s and increased in popularity in the 1980s as scholars in the social sciences began to recognize the ontological, epistemological, and axiological limitations of current research methodologies.^20^, ^21^ Rather than upholding a single, universal reality, these scholars sought to highlight the many complex personal realities of different groups and cultures through their research.^21^ Moreover, they acknowledged that the researcher's personal experiences and motivations shape the research process, challenging the idea that research is entirely neutral, impersonal, objective, and value‐free.^20^, ^21^ Methodologies such as autoethnography enabled these scholars to legitimize co‐constructed personal realities as valid forms of knowledge produced through rigorous research.^20^, ^21^ To date, autoethnography has been used to explore a variety of personal experiences and cultures, including but not limited to patient experiences,^22^ living with disabilities (e.g., dyslexia, dyspraxia),^23^, ^24^ clinical teaching,^25^ confidence,^26^ interprofessional education,^27^ and faculty development.^28^

One of the key benefits of this methodology stems from the dual role of the auto‐ethnographer as both researcher and participant. As an “insider”, the auto‐ethnographer has intimate, first‐hand knowledge of the personal or cultural experience in question.^29^ Moreover, they can provide insights into vulnerable moments or moments of everyday life that other research methods overlook.^20^, ^30^ In the autoethnographic process, the researcher produces and critically reflects upon “thick descriptions” of their personal experiences to weave a narrative that allows the readers to understand and empathize with the cultural group.^20^, ^21^, ^29^ The researcher then provides further context by grounding their experiences and interpretations in the existing literature.^21^ Moreover, the autoethnographic process can also be therapeutic for the researcher by giving them space to process, validate, and share their emotions and experiences.^21^ As a result, both reader and researcher can benefit from the insights gained through an autoethnographic study.

### Study purpose

Therefore, the purpose of this autoethnographic study detailing my first year as a faculty mentor in medical education is twofold:

1. Bring attention to the challenges that novice mentors face as they navigate their new professional identity and responsibilities, and
2. Provide recommendations on how to support novice mentors based on the challenges I encountered.

Through this autoethnography, I also hope to reflect on and process my own growth as a mentor and unique journey of professional identity formation while supporting fellow mentors who are navigating similar transitions in their roles and responsibilities.

## METHODS

### Reflexivity statement

#### My background and context

At the time of the study, I had completed approximately 5 months as a tenure‐track assistant professor at a large, research‐focused medical institution in the midwestern United States. In this role, I teach gross anatomy and neuroanatomy for medical, graduate, and anesthesiology assistant students as well as conduct medical education research. Besides anatomy education, one of my primary research areas includes metacognition (i.e., thinking about thinking), which provides me with the skills to critically reflect on and analyze my experience and performance. Prior to my faculty appointment, I earned a Ph.D. in Anatomy and Cell Biology (Education Track) and an M.S. in Modern Human Anatomy, both of which provided me with expertise in the theory and practice of anatomical education. Through these programs, I also collaborated with several excellent mentors who fostered my academic and professional development. These mentors allowed me the freedom to explore my academic interests, provided me with guidance and support, regularly communicated with me, valued my ideas and contributions, introduced me to new opportunities and collaborators, and were genuinely invested in my well‐being. Now, as a mentor, I strive to provide my mentees with the same quality of mentorship because I want to pay forward the kindness and support I once received to future colleagues. At the time, however, I had very little experience as a mentor, having only served as a research mentor for two high school students in a summer internship during my doctoral training. This early experience opened my eyes to the challenges of mentoring due to miscommunications and expectations between myself and the mentees. In my current position, I serve as a research mentor for multiple medical or anatomy education‐focused projects with medical (M.D.) and graduate (Ph.D. and M.S.) students both inside and outside my home institution.

#### My worldview

As a researcher, I hold a constructivist worldview and believe that individuals create reality through their unique experiences, perspectives, and interactions with the world. Rather than a single, objective reality, multiple realities can exist simultaneously, each equally complex and grounded in individual experience. From an epistemological standpoint, I believe that researchers and participants co‐construct knowledge and meaning as they navigate their lived experiences. Instead of passively identifying or interpreting themes that pre‐exist in the data, researchers actively generate them and give them meaning, thereby inserting themselves and their experiences into the process. As such, I will approach this autoethnographic study using a constructivist perspective and explore how I construct my professional identity as a mentor in medical education. As both researcher and participant, my lived experiences and interactions with the world will play a significant role in how I generate meaning from the data of this study.

### Data collection

The primary data source for this autoethnographic study included a series of audio diaries that I collected over a period of 8 months between January and August 2024. These audio diaries focused on events related to mentoring and my own development as a mentor, such as research project meetings with student mentees, mentorship trainings and workshops, and consultations with my own mentors. Each audio diary followed a semi‐structured format, which included the prompts^1^: What occurred in the event,^2^ how did it impact my view of mentoring, and^3^ what will I do differently in the future? To maximize recall of events and my own thoughts and emotions, I recorded the audio diaries as quickly as possible after the event (typically within 1 hour).

### Reflexive thematic analysis

Once data collection concluded, I transcribed the recorded audio diaries using Otter.ai (Otter.ai, Inc., Mountain View, CA), edited them for clarity, and imported them into Dedoose qualitative analysis software (version 9.0.62, SocioCultural Research Consultants, LLC, Los Angeles, CA). I then employed reflexive thematic analysis (TA) to generate themes from the audio diary data. This method of TA described by Braun and Clarke emphasizes the importance of the researcher and their unique philosophical sensibilities, theoretical assumptions, and thoughtful, reflective engagement with the data, which is aligned with my worldview and facilitates the creation of my unique reality through my experiences, perspectives, and interactions.^31^, ^32^ Reflexive TA includes six phases: (1) familiarization, (2) coding, (3) generating initial themes, (4) developing and reviewing themes, (5) refining, defining, and naming themes, and (6) writing up.^31^ Although Braun and Clarke outline a six‐phase process, they note that reflexive TA is flexible rather than prescriptive.^31^, ^32^ This flexibility meant I could freely shift between or blend phases during the analysis such as returning to add/discard codes or rename/redefine themes, which ultimately allowed me to be more thorough and precise in my analysis.^31^, ^32^

#### Phase 1: Familiarization

I began Braun and Clarke's reflexive TA by immersing myself in and becoming intimately familiar with my unique dataset.^31^, ^32^, ^33^ This familiarization occurred at two intervals: first, as I reviewed each diary transcript for accuracy and clarity, and secondly, as a stand‐alone activity after the transcripts were imported into Dedoose. During both pass‐throughs, I recorded memos of my initial impressions and insights on individual data files and across the entire dataset, which helped me develop a sense of the key ideas and patterns to be explored through the following phases of reflexive TA.

#### Phase 2: Coding

In the next phase of reflexive TA, I applied succinct labels (i.e., codes) to excerpts that represent a feature of the data that may be interesting or relevant to answering the research question.^31^, ^32^, ^33^ These excerpts included interactions with mentees, reflections on multiple rounds of coding the entire dataset occurred, followed by the collation of the codes and excerpts for subsequent organization and analysis.^32^, ^33^ Specifically with reflexive TA, coding can be flexible and allows for blending of multiple approaches.^31^ As such, I approached coding both deductively with codes based on TLT and inductively with codes generated from and unique to the dataset. My first round of coding started with codes based on each stage of TLT (e.g., *Disorienting Dilemma*, *Self‐Examination with Negative Emotion*, and *Planning New Course of Action*) and my initial memos. When I generated new codes mid‐round, I added them to the codebook and completed the current round of coding. I then applied the new code(s) to the earlier portions of the dataset in the subsequent round of coding. This process repeated until I no longer generated new codes.

#### Phase 3: Generating initial themes

This phase involves organizing the codes into related groups and patterns that serve as the initial themes.^32^ Some codes may represent overarching themes, others may be grouped into sub‐themes, and some may be discarded altogether. The discarded codes contained early ideas that either did not ultimately relate to mentoring or did not contribute to answering one of the research questions or to one of the generated themes. At this time, neither theme names nor definitions were generated, though some terms representing a concept were later carried over into the for definitive names and definitions (e.g., “communication”, “balance”). Although I performed the coding phase in Dedoose, I opted to conduct this phase on paper because it allowed me to visually arrange and rearrange the codes into themes and sub‐themes until I settled on a satisfactory structure.

#### Phase 4: Developing and reviewing themes

Afterward, I reviewed the provisional themes and sub‐themes against the coded excerpts in Dedoose to determine if they accurately represented the collected data, created coherent patterns of meaning, and formed a cohesive story that addressed the research questions. During this review, any themes that did not align with the coded excerpts or contribute to the research questions underwent further revision. This may have involved splitting, merging, rearranging, or discarding the themes and sub‐themes. The initial concept, idea, or pattern that served as the seed of the theme was then fleshed out more fully and given a more definitive name and definition, though it still underwent additional reorganization and renaming if needed.

#### Phase 5: Refining, defining, and naming themes

Once I confirmed that the provisional themes aligned with the data, I provided each with a name and definition that accurately represented the core idea and scope of the theme. These names and definitions underwent multiple rounds of revisions until they satisfactorily captured the essence of the theme.

#### Phase 6: Writing up

In this last phase, I compiled the finalized themes into a complete, analytic narrative that tells the story of the data and addresses the research questions. This write‐up is presented in the following sections of the article.

### Ethical considerations

As the primary participant, the Indiana University Institutional Review Board (IU‐IRB) determined that this research study did not require either exemption or approval. Nevertheless, I recognize that the information included in my reflections and in this autoethnography could potentially impact me or those that I interacted with during this study. To protect the privacy of my mentees and colleagues, all specific names, dates, locations, events, and project details were de‐identified and replaced with generic terms (e.g., “conference” or “project”). Given the sensitive and personal nature of some mentor‐mentee interactions, I also carefully selected the disclosed stories and excerpts to protect both myself and the mentees.

## FINDINGS

My first year as a faculty mentor was filled with a variety of new experiences, both positive and negative. During this period, I recorded 18 audio diaries encompassing mentor‐mentee meetings (*n* = 16), mentorship trainings (*n* = 1), and consultations with my own mentors (n = 1). In particular, the interactions with my mentees stirred a variety of emotions, ranging from pride, joy, gratitude, inspiration, and appreciation to frustration, disappointment, guilt, and stress. Although I encountered several disorienting dilemmas that prompted me to begin the steps of Transformative Learning Theory and develop my professional identity, I have yet to reach the end of the process. My disorienting dilemmas as a mentor have led me to self‐examine my abilities and assumptions as a mentor (Steps 2–4) and explore strategies for improvement (Steps 4–7), but I do not believe I have fully developed the confidence, competence, and practice of a successful mentor (Steps 8–10). These final steps will likely require additional time and experience to achieve.

### The balancing act

Through reflexive thematic analysis of my mentoring audio diaries, I identified the overarching theme of *The Balancing Act*, in which I felt I needed to constantly maintain my balance between an ever‐shifting collection of competing factors in my mentoring relationships. The data from the audio diaries suggest that finding a balance between factors such as time, effort, contributions, and expectations can lead to productive mentoring relationships, whereas failure to do so results in dysfunctional relationships. Included within this overarching theme are five sub‐themes: (i) *Balancing Project Contributions*, (ii) *Balancing Time and Obligations*, (iii) *Balancing Oversight and Autonomy*, (iv) *Shifting the Balance, and* (v) *Communicating the Balance*.

#### Balancing project contributions

During my doctoral studies, I became accustomed to performing much of the necessary groundwork of a research project myself. Tasks such as conducting literature reviews, collecting data, performing data analyses, and drafting manuscript were not only my responsibilities as a graduate student but also how I took pride in and ownership of my work. When I transitioned to the mentor role, I approached new projects with mentees in the same way, which led to some dissonance between my existing identity as a graduate student and my new role as a mentor. My mentees often expressed eagerness to complete these project tasks themselves, which led me to feel as though I had little to contribute. At the end of these project meetings, I frequently asked, “Is there anything you would like me to assist you with?” and my offers for assistance were regularly declined. Because I knew all too well how much time and effort were required to complete these foundational tasks to a high standard, I felt guilty for leaving so much work on the shoulders of the mentees:

When I'm collaborating with [mentees], and I don't have a very boots‐on‐the‐ground, hands‐on, role in a project, I feel like I'm not contributing to it. But that's not the case. I need to take a step back and recognize that as the mentor I'm not necessarily the one that's doing something to learn. I am providing the guidance on how to do something. It's a weird feeling that I just quite haven't gotten used to yet. I feel guilty for not being as hands‐on with every stage of the project like I'm used to as a graduate student.

This disorienting dilemma prompted me to reflect on why I felt guilty, which helped me reassess my professional identity and my role in projects now that I am the mentor rather than the mentee. As the mentor, I'm not necessarily responsible for moving a research project forward. Rather, I am responsible for ensuring that my mentee has the support and guidance they need to move the project forward themselves. This allows the mentee to gain first‐hand experience in conducting research and critically thinking through challenges. If I completed most of the project as I did as a graduate student, the final product may be better, but I would detract from the mentees' learning experience:

I wasn't teaching this person anything by backtracking and redoing it for the sake of accuracy and making sure it was done right, rather than letting them do it and then teaching them how to improve.

Moving forward, I need to transition away from my identity as a graduate student and find a balance in both what I view as contributions to a research project and how those contributions are divided between myself and the mentee. Thinking back, my own mentors provided guidance on both the “what” and the “why,” but they entrusted me with completing tasks on my own. Although I may not have my metaphorical “boots on the ground” with every aspect of future projects anymore, I'm no less a contributor. Instead, my contributions come in the form of expertise, guidance, and support for the mentee:

I'm contributing the expertise, the experience, the knowledge that the student didn't really have and was looking for in this meeting. This kind of alleviated some of that discomfort I had in previous meetings with students about not doing the legwork with them on a project. [The mentee] mentioned in the meetings, like, “oh, I've never done research before, so thank you for explaining this to me”. It made me feel like I was contributing to a project, just in a different way. Like this was something that my old mentors would do. After I wrote a draft, they would review it. It's not like they were sitting next to me, writing the different sections. They would review it, give me critiques, and give me advice afterwards. This really felt like I was being a mentor and being a mentor in the right way, and it felt comfortable.

Furthermore, I should not automatically feel guilty for “burdening” the mentee with the responsibility of these tasks. In many cases, students eagerly undertake these tasks to take ownership over the project as I once did. Rather, I should consider the desirable difficulty of the tasks and what the mentee stands to learn from completing the task.

#### Balancing time and obligations

As I began working with multiple mentees, I found it increasingly difficult to balance my responsibilities as a mentor and my own academic responsibilities. Though mentoring students was a rewarding and enlightening experience, it required significantly more time and effort than I had initially anticipated. As the mentor, I held regular project meetings, taught new concepts and skills, reviewed submitted work, and provided constructive, individualized feedback to support the progress of mentees, but this often limited my own ability to complete my other academic responsibilities. Rather than managing the one or two projects I had as a graduate student, I now juggled four or five at a time. This became particularly difficult and stressful when my mentees and I needed to meet strict deadlines such as conference abstract or graduate credit deadlines. In these situations, I struggled to balance efficiency with learning experience. On the other hand, I could complete a particular task much more quickly myself to meet a deadline, but it would detract from the mentee's learning experience. Conversely, allowing the mentee to undertake the task independently, make mistakes, and revise based on constructive feedback resulted in a high‐quality, albeit time‐consuming learning process:

I keep having to remind myself that the work the student is doing is part of the learning experience, and if I take over those tasks and just do them quickly myself without letting the student see how I'm doing it, explaining it to them, or giving them an opportunity to do it in the first place, they aren't learning anything. They're just a passenger along for this ride.

This disorienting dilemma prompted me to reflect and reassess how to best balance my own responsibilities and my responsibilities to my mentees. Shifting my project contributions from foundational tasks to a feedback and support role—and allowing the mentee to determine how much they want to take on at a time—has helped alleviate this issue to some extent. However, I still need to continue improving in this area as I take on new teaching, research, and administrative responsibilities in my appointment.

#### Balancing oversight and autonomy

Balancing my approach to mentoring between too little oversight and too much micromanagement also proved difficult in my first year as a mentor. As a graduate student, I appreciated when my mentors gave me the freedom to complete assigned tasks in my own time and manner, rather than micromanaging every aspect. This approach allowed me to develop my own workflow and to learn how to find answers independently. As a mentor, I wanted to afford my students the same freedoms and opportunities. However, I quickly realized that this approach did not meet the needs of all my mentees.

For example, one disorienting experience that caused me significant stress and anxiety occurred when a mentee and I planned to submit a poster for an upcoming conference. We had discussed the deadline, but I remained relatively hands‐off, allowing them to work at their own pace and reach out for assistance as needed. As the deadline approached, I became increasingly concerned that the mentee would not complete the poster to the appropriate standard in time. After several friendly reminders, I wondered if and when it would be appropriate for me to step in and complete the poster on their behalf to meet the deadline. I also reflected on where I may have gone wrong and how I could have avoided this situation by either scaffolding the process of creating the poster, checking in more frequently, or some other approach:

So getting closer to the deadline, the same situation happened where it was getting really, really close to the end, but the student who otherwise would know how to do these things wasn't getting it done by that deadline. And so it got to the point where, like, should I step in? Should I help out? Should I do more of [these tasks]? I don't particularly want to just because I got a lot of other things going on, but at the same time, like, I don't want this person to fall flat on their face. I don't want them to, but at the same time, I don't know if that's part of the learning experience to kind of let that happen.

Through this experience, I learned that I need to balance oversight and autonomy as a mentor. Whether due to inexperience, lack of motivation, working style, or some other academic or personal factor, some mentees benefit from more direct oversight and guidance, whereas others would benefit from greater autonomy and freedom to learn independently.

In this case, we were a lot more of a team working on a particular project, versus in the other case, it was more of, I guess, assigning tasks to a mentee. It doesn't necessarily mean that either one is bad. Sometimes there's a lot of both, like if one mentee is a little bit more inexperienced in an area, there's going to be a lot more of assigning tasks and giving them directions on how to do things, versus someone who may be a little bit more experienced or a little bit more self‐driven. It's more of a collaborative role where you're discussing things and coming to a conclusion together, versus assigning them tasks.

However, the ideal balance for each mentee remains elusive. Moving forward, I still need to identify the ideal balance between too much trust and autonomy and too much oversight and micromanagement.

#### Shifting the balance

Although I learned that effective mentorship required a balance between multiple factors, I continued to struggle with achieving the necessary balance for each mentee. Each mentee needed support in different ways, meaning the balance I achieved with one mentee would not necessarily translate to another mentee. As such, I felt like I had to constantly shift my balance with each mentee interaction and stay cognizant of each mentee's needs:

It feels like a moving balance, or a shifting balance, that as a as a mentor, I need to be cognizant of and shift my weight from one side, like, “Okay, I need to give more direction to this person, because they're inexperienced”, and then immediately going over to the next meeting with someone more experience, I have to, like, shift my weight to the opposite side and step back and not really give directions too much, but let this person actually take lead. It's like I'm standing on a plank that's balanced on a barrel and I have to shift my balance back and forth.

This constant shifting proved rather difficult and frustrating for me since I already struggled with finding the appropriate balance for a single mentee let alone multiple. However, I felt I needed to master this constant flexibility as a mentor, considering that all mentees have differing needs. As a mentor, I won't always have the option to decide who I work with, so I'll need to adapt and balance my mentorship accordingly:

I honestly don't know which side of the spectrum I would work well with at this stage since I am pretty new to mentoring. I guess it'll be a trial‐and‐error process, and to some extent, I feel like I have to be good at working with both, because I'm not always going to have a mentee that's going to fit very, very well with me. It's not always going to be a perfect one‐to‐one match on all qualities and traits. So moving forward, when I do actually start to interact with these students, something that I'm going to have to be aware of is that this is going to be a learning experience, and I'm going to have to figure out how to change my mentoring style and how to work with these mentees depending on their specific needs.

#### Communicating the balance

Although finding and maintaining the ideal balance while mentoring can present quite a challenge, communication greatly reduces the difficulty. In my limited experience as a mentor, open, regular communication with mentees about expectations, needs, and goals helped immensely because it allowed me to adjust my mentoring style to better support the mentee. Once I realized the mentee's needs, I could adjust more readily to support them with increased direct guidance, autonomy, and support, rather than trying to figure out the balance myself (and potentially over‐stepping). Conversely, the mentee also better understood what they needed to accomplish.

It just comes down to greater communication between both parties, understanding each other's working styles, and for me specifically, finding a little bit more of a balance between whether I should step in or not. And the weird thing is, I feel like it's going to be different for every scenario. So it's a balance, but at the same time, it's a moving balance.

However, when the lines of communication collapse, maintaining the balance becomes much more challenging. The mentoring relationships in which I struggled to find a balance often lacked communication, either on my part, the mentee's, or both. In hindsight, many of the obstacles and frustrations that arose could have been mitigated with more open, consistent communication of expectations, goals, and needs.

## DISCUSSION

Transitioning from mentee to mentor can present a significant challenge for inexperienced faculty mentors. Through this process, I learned that becoming an effective mentor requires a delicate balance in several areas, including how I contributed to projects, how I managed my time and responsibilities, and how I supervised my mentees. I also found that the balance I needed to maintain changes with each mentee, requiring me to shift my attention between their diverse expectations, needs, and goals. These imbalances frequently caused stress and anxiety, prompting me to reflect on my professional identity and rethink my approach to mentoring. Although this dynamic balancing act posed a major challenge, I found that open, consistent communication between myself and the mentee allowed me to adapt my mentoring style to better support their development. This autoethnographic journey has taught me a great deal about what it means to be an effective mentor and has helped shape my emerging mentor identity. Still, though, I recognize that I have much more to learn.

Early into my first year, I struggled with how I should meaningfully contribute to research projects in my new role. Previously, as a graduate student, I took great pride in independently completing the main aspects of a research project. However, when I began delegating these tasks to my mentees, I felt guilty and unproductive. This likely occurred due to dissonance between my well‐established and emerging professional identities. At the time, I had only just started shaping my professional identity as a mentor and had few life experiences and limited socialization to base it upon. However, when I reflected on how my mentors outlined the “what” and the “why” but entrusted me with completing the tasks independently, I felt reassured that this approach to mentoring was appropriate. As I continued to serve as a mentor, I began feeling more comfortable relinquishing tasks to students and relying on them to drive projects forward while I provided guidance and support. This suggests that my professional identity began evolving and I started to normalize these previously uncomfortable behaviors. As I continue to serve in this role, I anticipate my mentor identity will solidify further with additional experience and socialization.

In hindsight, my early struggles with my professional identities would have been smoother had I consistently consulted my network of peers and mentors. Interactions with role models, mentors, and peers play a critical role in professional identity formation.^13^, ^14^, ^18^ Through these relationships, I could have received additional guidance, support, and reassurance for navigating the mentor role, especially in difficult situations.^13^, ^14^, ^18^ This likely would have eased and expedited my transition into the mentor role. As such, I recommend that other novice faculty mentors not only identify experienced mentors and peers they trust but also regularly consult them to support their professional identity formation.

Through mentoring, I also discovered the significant influence time has on how faculty members choose to mentor trainees, if at all.^3^, ^4^, ^5^ Due to time constraints, I found myself balancing productivity against the mentee's learning experience, and debating if and when I should step in to complete a task to maintain adequate progress or meet deadlines. This internal conflict stemmed from my concerns about maintaining adequate research productivity and that poor quality work reflected poorly on my potential as a researcher and mentor. As I started taking on more mentees, the time and energy I could commit to each reduced even further, exacerbating this internal conflict. After overextending myself, I reflected on my mentoring priorities. Although I greatly appreciated the assistance in moving projects forward, I wanted to support students as I was supported in the past. In the future, I intend to mentor only when I can commit the necessary time and energy to support the growth and development of the mentee, rather than prioritize the results they produce.

Several previous studies have similarly found that faculty often decline to serve as mentors due to teaching, research, and service commitments that compete for their limited time and attention.^3^, ^4^, ^5^, ^34^ Instead, faculty prioritize endeavors that institutions value and incentivize, such as research productivity and grant funding.^3^, ^7^ Nevertheless, faculty often serve as mentors not for the research or career benefits, but rather a personal sense of duty or for personal satisfaction and fulfillment.^7^ However, faculty members who take on too much mentoring before establishing their own positions risk stalling their own professional development, to the detriment of themselves and their mentees.^4^ To avoid the negative effects of overextending for both the mentee and mentor, I recommend that novice faculty mentors first assess whether they have the time, energy, and resources to commit to mentorship. It is also important to reflect on their motivations for taking on the role. Authentic mentorship demands a significant investment, and as I have learned, stretching oneself too thin can result in a frustrating and negative experience for everyone involved.

My first year as a mentor also impressed upon me the importance of tailoring my mentoring style to the needs of each mentee. Both in my own experience and according to the literature, mentees can vary greatly in their needs and goals.^3^, ^4^, ^35^ In particular, I found that I needed to regularly adjust the degree of direct oversight or autonomy I provided each mentee. Some experienced or self‐motivated mentees thrived with greater autonomy, whereas mentees who were inexperienced or procrastinated often benefited from more consistent oversight. Although I found this constant shifting challenging, I believe it is essential to ensuring that each mentee is supported as much as possible. Some veteran mentors also believe the ideal approach involves tailoring to the mentees and meeting them where they are initially.^9^ As the mentoring relationship progresses and the mentee develops their skills, the mentor can then shift their balance and progress toward granting greater autonomy and entrustable tasks.^35^ In the case of mentees who struggle to progress and the balance remains stagnant, the mentor can create momentum by sharing their own passion for the research and scaffolding the larger research project into smaller tasks, thereby setting the mentee up for small victories.^9^ Regardless of the starting balance of the mentee, the mentor still needs the ability to shift their mentoring style—their balance—both as a mentee develops and as the mentor switches between mentees.^3^, ^4^, ^12^, ^35^ This can be a challenging endeavor, especially for novice mentors.

Through my mentoring experience, I have found clear, regular communication with the mentee about their needs, expectations, and goals to be instrumental in efficiently finding and shifting my mentoring style accordingly. This perspective aligns with the mentorship literature, which agrees that consistent communication plays a critical role in a successful mentoring relationship.^1^, ^3^, ^4^, ^9^, ^35^ Communication skills such as active listening, direct language, rephrasing statements, and conflict resolution can facilitate effective communication between mentor and mentee.^1^, ^3^, ^4^, ^35^ With open communication, mentees feel more comfortable vocalizing their needs and expectations, as well as contributing their own ideas to a project.^3^ Conversely, frequent miscommunications often indicate that one's mentoring skills need refinement.^3^ To avoid miscommunications, novice mentors should be mindful of how they communicate, actively develop their communication skills, and maintain open conversations with their mentees. For example, self‐assessment instruments such as the Thomas‐Kilmann Conflict Mode or Dominant, Influencer, Steady, and Conscientiousness (DISC) Instruments can help mentors and mentees determine the strengths and weaknesses of their communication styles and address them accordingly.^36^, ^37^ Mentoring agreements in which both the mentor and mentee explicitly outline and align goals and expectations can also help prevent miscommunication.^38^, ^39^ I intend to incorporate these elements into my own mentoring in the future, and I recommend other novice mentors do so as well to enhance communication and more readily adapt to mentee needs.

## LIMITATIONS

In this autoethnographic study, I explored my experiences as a first‐year faculty mentor from my own unique worldview. However, in keeping with constructivist principles, other individuals, including those who participated in the “same” event as me (e.g., mentees or mentors), may have different, equally valid interpretations of the events due to their own unique worldviews. I include this as a limitation not because it diminishes the value of the insights included in this study, but rather to acknowledge the kaleidoscope of mentoring perspectives and experiences that remain unexplored. Additional qualitative studies should be performed to capture these unique, differing perspectives in the future.

Another limitation of this study stems from the logistics of the audio diaries. For practical reasons, the audio diaries were only recorded after mentor‐mentee meetings, mentorship trainings, and consultations with my own mentors. They did not include all the brief, impromptu mentoring moments that occurred, such as e‐mail correspondence or unexpected hallway chats, due to constraints on time, location, or privacy. Given that mentoring can be both formal and informal, and occur during both planned and unplanned moments, the omission of these moments may have resulted in the loss of some insight into my development as a mentor. Moreover, due to the low number and quality of audio diaries recorded after mentorship trainings and mentor consultations (*n* = 1 each), I could not generate any themes related to these specific events. The two diaries were relatively brief (~5 min long) and did not include the same thoughtful reflection in which I question my actions, my perspectives on mentoring, or how I should have or will approach mentoring in the future. Rather, they include more superficial summaries of events that transpired. Despite these omissions, this autoethnographic study still includes several audio diaries with thick descriptions that capture my mentoring experiences.

Lastly, although my expertise and training are in the anatomical sciences, many of the projects I collaborated on with mentees related more broadly to medical education and science communication. As such, the conclusions from these experiences likely apply to mentoring in the anatomical sciences but fail to capture the unique nuances of mentoring within this specific field. For example, working with full body donors in the human anatomy lab presents unique opportunities to mentor students on professionalism, ethics, empathy, respect, death, and emotions.^40^, ^41^, ^42^ A future autoethnographic study could explore the experience of mentoring learners or junior anatomy educators in these principles in the context of the human anatomy lab.

## CONCLUSIONS

As I reflect on my first year as a faculty member and research mentor, I am thankful for the mentoring experience I gained in the process. Though I encountered disorienting dilemmas that resulted in stress, anxiety, and guilt, these events pushed me to reflect and reexamine how I approached mentorship. As a result, my professional identity began evolving, and I gained a greater appreciation for the balancing act that is mentorship. To be an effective mentor, one must continuously balance several factors, such as project contributions, time, resources, and supervision based on the progress, needs, and goals of their diverse mentees. Although I am now more cognizant of these challenges, I still have significant room for growth as a mentor.

## AUTHOR CONTRIBUTION

**Andrew S. Cale:** Conceptualization; methodology; data curation; project administration; writing – review and editing; writing – original draft; investigation; formal analysis.
